# Role of gut microbe-derived metabolites in cardiometabolic diseases: Systems based approach

**DOI:** 10.1016/j.molmet.2022.101557

**Published:** 2022-07-21

**Authors:** Yang Cao, Ruben Aquino-Martinez, Evan Hutchison, Hooman Allayee, Aldons J. Lusis, Federico E. Rey

**Affiliations:** 1 Departments of Medicine, Human Genetics, and Microbiology, Immunology, & Molecular Genetics, David Geffen School of Medicine of UCLA, Los Angeles, CA 90095, USA; 2 Department of Bacteriology, University of Wisconsin, Madison, Madison, WI 53706, USA; 3 Departments of Population & Public Health Sciences and Biochemistry & Molecular Medicine, Keck School of Medicine, University of Southern California, Los Angeles, CA 90033, USA

**Keywords:** cardiometabolic diseases, systems genetics, gut microbiome, metabolomics

## Abstract

**Background:**

The gut microbiome influences host physiology and cardiometabolic diseases by interacting directly with intestinal cells or by producing molecules that enter the host circulation. Given the large number of microbial species present in the gut and the numerous factors that influence gut bacterial composition, it has been challenging to understand the underlying biological mechanisms that modulate risk of cardiometabolic disease.

**Scope of the Review:**

Here we discuss a systems-based approach that involves simultaneously examining individuals in populations for gut microbiome composition, molecular traits using “omics” technologies, such as circulating metabolites quantified by mass spectrometry, and clinical traits. We summarize findings from landmark studies using this approach and discuss future applications.

**Major Conclusions:**

Population-based integrative approaches have identified a large number of microbe-derived or microbe-modified metabolites that are associated with cardiometabolic traits. The knowledge gained from these studies provide new opportunities for understanding the mechanisms involved in gut microbiome–host interactions and may have potentially important implications for developing novel therapeutic approaches.

## 1 Introduction

The gut microbiome plays a critical role in host metabolism, influencing traits such as obesity, diabetes, and other disorders. Because the microbiome is highly complex, with hundreds of species, and because its composition is influenced by many factors (including the host's diet, age, genetics, health, and lifestyle), it has been challenging to determine the mechanisms involved in microbiome–host interactions. Here, we discuss population-based approaches that are proving useful in understanding the molecular mechanisms mediating the effects of the microbiome on cardio-metabolic disorders. Particularly informative have been studies that integrate global “omics” data with physiological/clinical data in human or mouse cohorts to help understand mechanisms underlying complex traits such as cardiovascular disease. For example, gut microbiome composition obtained by sequencing can be integrated with metabolomics data in the host to identify which bacteria or metabolites mediate physiological microbe-host interactions. If host genetic data are incorporated into the analyses, the approach has been termed “systems genetics” (Box 1). Such data can be integrated using genetic mapping, correlation, or various types of modeling. Thus far, the most significant progress has come from integrating variation in microbiome composition, plasma metabolites, and clinical traits. The first section provides an overview of microbial metabolism in the gut. The second section discusses the environmental and genetic factors influencing gut microbes and bacterial-derived metabolites. The third section reviews our current understanding of microbe-derived metabolites involved in cardiometabolic diseases. The last section summarizes findings and discusses future prospects.

Box 1 **Systems genetics.**

**Figure 1:**
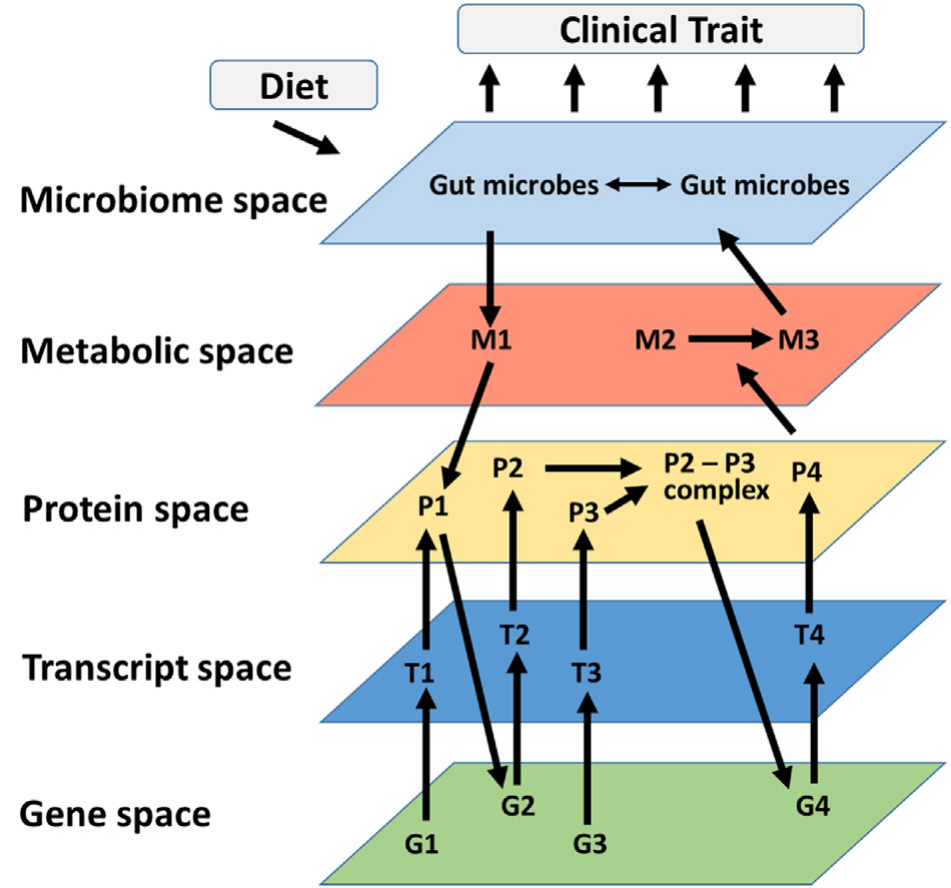
**System genetics strategies.** Molecular phenotypes across multiple biological scales — including the genome (G), transcripts (T), proteins (P), metabolites (M) and microbiome — are examined using “omics” technologies in populations exhibiting natural variation. Interactions (shown as arrows) can then be identified using genetic mapping, correlation structure, causal modeling, and network modeling. For example, based on natural variations of genes 1–4 (G1–4), interactions between the genes and related molecules can be modelled. These molecular interactions are then examined in the context of clinical variation in the population. Modified from Civelek and Lusis, Nat Rev Genet 2014.

Reductionist experimental approaches are powerful in that they can establish causality. However, because such studies generally examine one element at a time, and usually study perturbations on a single genetic background, the conclusions that can be drawn concerning the interactions that occur in complex traits and diverse populations are limited. By comparison, “systems genetics” is a population-based approach that can address such complexity by integrating molecular and clinical variation among individuals that differ in genetics and environmental exposure. Systems genetics uses high throughput “omics” technologies, such as DNA sequencing, RNA sequencing (RNAseq), metabolomics, or proteomics, to quantitate molecular phenotypes alongside clinical phenotypes in populations of humans or experimental organisms (Figure 1). The underlying concept is that genetic and environmental variations affect complex clinical traits by perturbing molecular traits, such as gene expression or metabolite levels, and thus by measuring these as a function of genetic and environmental variation among in individuals in a population, one can understand their relationships [1,2].

Alt-text: Box 1

## 2 The role of gut microbes (direct) and related metabolites (indirect) in normal physiology and complex diseases

Mammals harbor diverse and dynamic microbial communities in their intestines that play key roles on host biology, including maturation of the immune system, breakdown of complex dietary components, vitamin production, energy harvesting, drug metabolism, and protection against enteric pathogens. For example, germ-free (GF) mice are protected from developing diet-induced obesity and insulin resistance [[3], [4], [5]] have decreased levels of TNF-a in serum, and have increased lipid excretion relative to age and sex matched conventionally-raised animals [6]. A foundational study from Bäckhed and colleagues further demonstrated that gut microbes increase both nutrient absorption and hepatic lipogenesis, with colonized mice accumulating 60% more fat tissue than GF counterparts despite lower caloric intake [3].

Gut microbes can also affect host physiology through production of metabolites that act on specific receptors expressed in the intestine and in distant organs, impacting multiple cellular signaling cascades and causing epigenetic changes [[7], [8], [9], [10], [11], [12]]. As discussed in further detail below, these metabolites are derived from various classes of dietary nutrients, including quaternary amines, amino acids, and carbohydrates (Table 1). Myriad effects related to gut and systemic physiology and cardiometabolic disease have been described for these molecules [13,14]. These paracrine and endocrine effects of gut microbial metabolites constitute a direct link between environmental exposures and host cellular function [15]. Furthermore, environmental exposures including Western-like diets, antibiotics, stress, and aging often lead to changes in microbial metabolism and production of metabolites or toxins that contribute to disease states [16,17].

**Table 1:** Characteristics of gut bacteria-derived metabolites associated with cardiometabolic traits.

Precursor or dietary nutrient	Metabolite	Target tissue	Biological Pathway(s) Affected
Quaternary amines			
Choline, carnitine	TMAO	Liver Vessel wall Platelets	Adverse effects on lipid/glucose metabolism Promotes inflammation/oxidized LDL update Activates and promotes thrombosis
Amino acids			
Tryptophan	IPA	Intestine	Improves barrier function
		Pancreas	Improves beta-cell function
		Liver Heart Vasculature	Decreases lipid content, steatosis, inflammation, fibrosis Improves contractility and mitochondrial function Increases blood pressure
Phenylalanine	PAG	Platelets	Activates and promotes thrombosis
Histidine	Imidazole propionate	Liver, skeletal muscle, adipose Heart	Impairs Insulin signaling
Tyrosine	Phenols	Kidney Intestine	Reduces kidney function Reduces epithelial barrier function
BCAAs	BCAAs	White adipose, skeletal muscle, liver, heart, kidney, and pancreas	Increases insulin resistance
Microbiota-accessible carbohydrates (MACs)	Butyrate	Intestine Regulatory T cells	Improves barrier function Decreases inflammation
	Acetate	Liver	Attenuates steatosis and inflammation
	Propionate	Intestine	Reduces cholesterol absorption and atherosclerosis
Sterols			
Primary BAs	Secondary BAs	Liver, intestine, pancreas, adipose	Improves glucose homeostasis and energy expenditure

Direct interactions between microbes or microbial components and the host also have major implications for host health. In this context, the intestinal epithelium constitutes a dynamic interface between the gut environment and the host. Acting as a physical and immune barrier, the gut epithelium plays an essential function to retain water and absorb nutrients, while avoiding microbial invasion [7,18]. Epithelial cells are covered by a mucus layer that contains digestive secretions, immune molecules, antimicrobial peptides, and cytokines, limiting the contact between gut microbes and luminal antigens with host epithelial cells [19]. Together, the epithelial cells and their tight junctions, which help seal off the epithelium, the mucus layer and the antimicrobial peptides contained within play a critical role in preventing trespassing microbes and their potentially harmful toxins from reaching underlying tissues. Thus, gut barrier integrity is critical in the prevention of intestinal and extraintestinal disorders. In this regard, human studies have consistently reported that changes in gut microbiome composition are associated with altered intestinal barrier function and increased circulating LPS levels [20,21]. While translocation of microbial components at low levels is key for priming innate defenses, chronic systemic inflammation induced by translocation of bacterial cellular components, including LPS and peptidoglycan fragments, is a potential mechanism by which gut bacteria contribute to cardiometabolic diseases [21].

## 3 Factors affecting gut bacterial composition

### 3.1 Environment

A large number of environmental factors, including delivery mode and diet, can influence the composition of the gut microbiome. Children that are delivered naturally, rather than by Caesarean section, tend to have more diverse gut microbiomes early in life [22]. Antibiotics cause large changes in the gut microbiome, although the degree of resulting perturbation is determined by many factors including age, duration of treatment, spectrum, and treatment route. Additionally, post-antibiotic environment including exposure to pathogenic and beneficial microbes impact microbiome composition after treatment [23]. Recently, pollutants and smoking have been shown to have an impact on gut microbiome composition [24]. The gut microbiome also changes as people age and microbiome signatures have been associated with healthy aging [25,26]. Furthermore, dietary interventions rich in non-digestible carbohydrates modulate the gut microbiome of old adults and have been associated with healthy aging [27]. Non-digestible carbohydrates are considered a major source of carbon and energy for intestinal microbes [28] and these can have a major impact the composition of the microbiome [29]. Dietary proteins are an important nitrogen source for bacteria [30], whereas fat, while generally considered to be less important to distal gut microbes, can still modulate microbiome composition [31,32].

### 3.2 Host genetics

Human and animal studies have provided evidence that host genetics also plays an important role in determining the composition and function of the gut microbiome. For example, family- and population-based analyses have yielded significant heritability estimates for a sizeable fraction of bacterial taxa [33]. Among a panel of 110 inbred mouse strains from the Hybrid Mouse Diversity Panel (HMDP), where confounding factors, such as dietary variation and age were controlled, heritability estimates for certain taxa were considerably higher than in humans [34]. Consistent with these observations, dozens of loci associated with gut bacterial abundance have been identified through genome-wide association studies (GWAS) in both mice and humans [[34], [35], [36]]. However, most loci have not been replicated across studies, at least in humans, even with sample sizes exceeding 1000 subjects [33,[37], [38], [39], [40], [41], [42], [43], [44], [45], [46]]. The only two exceptions thus far have been the *LCT* and *ABO* loci, which are the basis for the lactase persistence trait and ABO blood group system, respectively. Across studies, the lactase non-persistence genotypes at *LCT* were associated with higher intestinal levels of bifidobacteria, which are able to break down lactose [47], and one study further provided evidence that the lead variant at *LCT* was causally associated with multiple dietary and metabolic phenotypes, including obesity and type 2 diabetes (T2D) [46]. By comparison, taxa associated with *ABO* were not the same across the three datasets in which this locus was identified [40,41,43]. A study using microbiome data from 1,046 healthy individuals showed that environmental factors dominate over host genetics in determining gut microbiome composition [42]. As the field moves towards larger sample sizes and metagenomic sequencing (vs. 16S rRNA gene sequencing), association signals for gut bacteria abundance will be better refined. Furthermore, heritability estimates and genetic studies can be expanded to functional components of the gut microbiome, which may reveal another level of insight into how host genetics influences susceptibility to cardiometabolic diseases.

Another important aspect of host genetics is sex differences. It has proven difficult to demonstrate sex differences in gut microbial composition in humans, undoubtedly due to the impact of environmental and other factors, but sex differences are clearly observed in mice. For example, gut microbiota in the HMDP discussed above were studied for sex differences in composition [48]. A total of about 700 male and female mice from 89 matched strains were examined using 16S rRNA gene sequencing. When considering each strain independently, clear differences in microbiota composition and diversity were observed between sexes. Using PROCRUSTES analysis, the magnitude and direction of change for multiple bacterial species were different between strains, suggesting that the impact of sex depends on host genotype. However, when an entire mouse population was examined together using Bray–Curtis dissimilarity, no clear pattern differentiating males from females were observed. Thus, genetic variation between strains obscures sex differences when the entire population is examined together. Follow-up studies in a small number of strains showed that the sex differences were at least in part mediated by sex hormones and these sex differences were associated with differences in bile acid metabolites [48].

## 4 Gut microbe-derived metabolites and cardiometabolic disease

**Figure 2:**
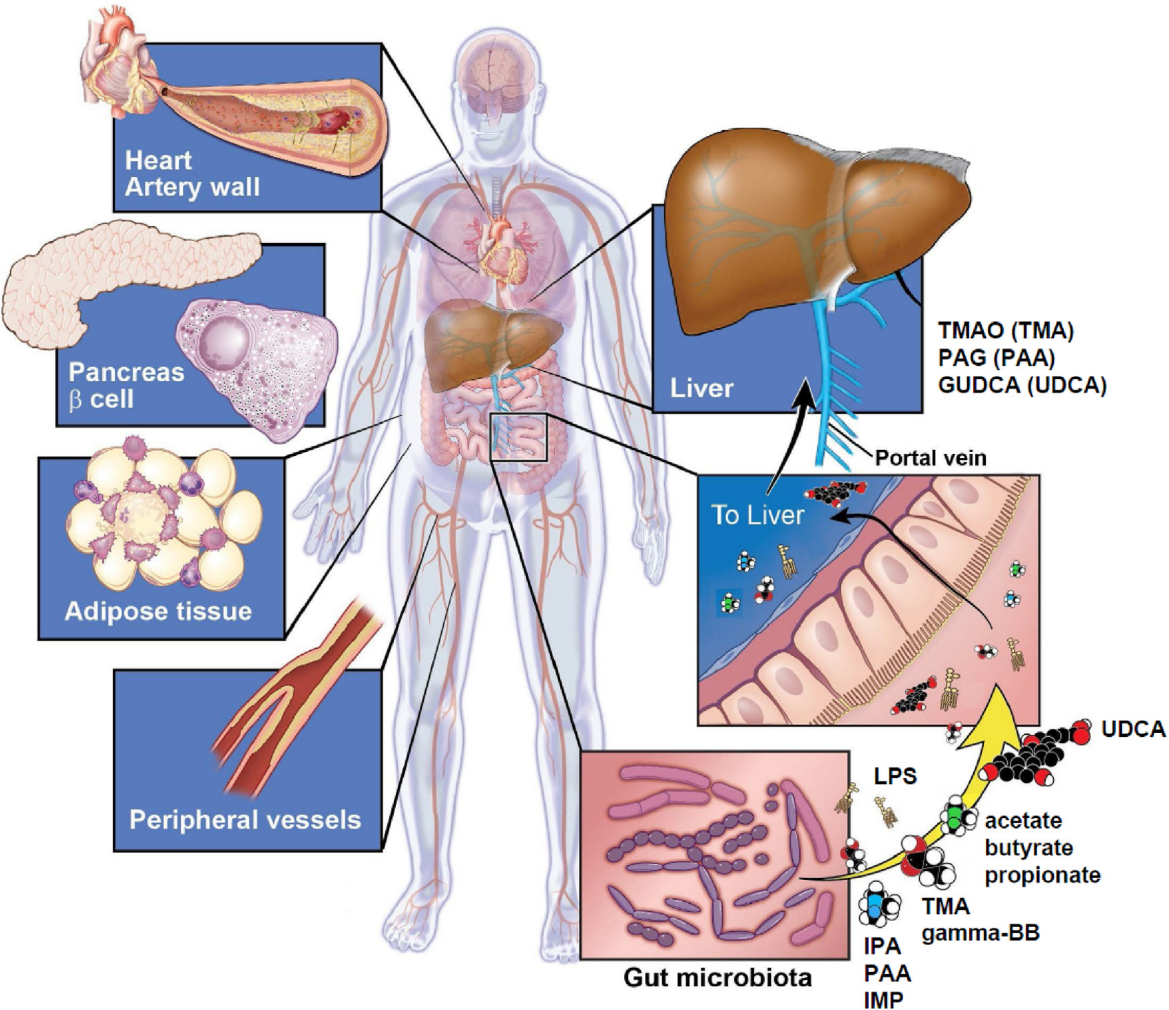
**Gut microbiome-host interactions through metabolites**. Alterations of the gut microbiome can impact development of cardiometabolic phenotypes through biological effects in coronary and peripheral vessels, liver, adipose, and pancreas. These effects can be mediated SD through intermediates generated by gut bacteria from nutrients, such as amino acids (IPA, PAA, IMP), quaternary amines (TMA, gamma-butyrobetaine), and non-digestible carbohydrates (acetate, butyrate, propionate) or host-derived compounds (i.e., GUDCA) that are metabolized by microbes (i.e., UDCA). In some cases, the intermediates (e.g., TMA or PAA) are absorbed and further metabolized by the host to produce bioactive metabolites, such as TMAO or PAG.

A number of gut bacteria-derived metabolites have been implicated in the development of cardiometabolic outcomes. In some cases, various dietary nutrients serve as the exogenous precursors that gut bacteria use to generate the metabolite in question. By contrast, metabolites first generated by the host can also be acted upon by gut bacteria, particularly those that are hydroxylated or otherwise modified for excretion through the kidney. A discussion of each of these classes of metabolites and their relationships to gut bacteria and cardiometabolic outcomes is provided below (Table 1 and Figure 2).

## 5 Metabolites derived from quaternary amines (choline/carnitine/TMAO)

Choline is an essential nutrient required for diverse biological functions ranging from synthesis of acetylcholine, a membrane component, to cell membrane signaling [49]. In humans, endogenous choline production is insufficient, and dietary sources are needed to prevent its deficiency [50]. In early life, the beneficial effects of choline are associated with the neurological health of the child during pregnancy [50], where maternal supplementation with dietary choline supports brain development, provides neural protection against metabolic and stress insults, and improves cognitive function [50].

More recently, choline was also recognized as a substrate for gut bacterial metabolism (Table 1). Wang and colleagues [17] carried out plasma metabolomics on a cohort of patients with and without coronary artery disease (CAD) and identified higher circulating levels of choline and the metabolite, trimethylamine *N*-oxide (TMAO), as being associated with atherosclerosis [17]. TMAO is the product of hepatic oxidation of the microbe-derived metabolite trimethylamine (TMA), which itself is generated from dietary choline by gut bacteria harboring glycyl radical enzyme choline TMA-lyase (CutC) [51]. TMA is rapidly absorbed from the intestine into the portal system and oxidized by flavin monooxygenases, primarily FMO3, into TMAO [52]. Notably, TMAO can also be produced from dietary carnitine (Table 1), through either a minor route where TMA is directly generated from carnitine [53,54] or a major route involving two sequential bacterial reactions; the first generates gamma-butyrobetaine (γbb), which is then converted to TMA by the enzymes encoded by γbb utilization (bbu) gene cluster. Several host-associated bacterial species from the order Clostridiales encode *bbu*-like gene clusters including *Emergencia timonensis* [[54], [55], [56]]. In either case, subsequent studies confirmed the proatherogenic effects of choline- or carnitine-derived TMAO on aortic lesion development in mouse models through pro-inflammatory and pro-coagulant effects [53,57]. In addition to atherosclerotic pathologies associated with TMAO, the FMO3 enzyme has also been associated with metabolic disturbances, such as altered lipid and glucose metabolism [[58], [59], [60], [61]]. Interestingly, insulin resistance increases FMO3 activity in the liver of both human and mice, which leads to increased TMAO levels and metabolic dysfunction over time [58]. This may be related to the specific activation of endoplasmic reticulum stress kinase (PERK) by TMAO but not by choline or carnitine [62].

Gnotobiotic mouse studies suggest that exacerbated gut bacterial choline metabolism can influence choline bioavailability. Romano and colleagues identified members of the human gut microbiota that transform choline into TMA, leading to decreased choline availability to the host [63], which recapitulated aspects of diet-induced choline deficiency when the choline consuming bacteria represented a significant fraction of the microbiome [64]. Consistent with the role of choline in neural development, exacerbated maternal gut microbial metabolism of choline into TMA can alter brain development and influence behavior in the offspring potentially by decreasing sphingomyelin and acetylcholine levels [64]. It is important to note that these studies were done using diets supplemented with free choline, which can be readily degraded under anaerobic conditions by gut microbes. However, the most abundant form of dietary choline is phosphatidylcholine, which is less available to TMA-producing species as the choline has to be hydrolyzed. Chittim et al. demonstrated that gut bacteria can hydrolyze phosphatidylcholine and release choline via phospholipase D enzyme and influence TMA production and potentially TMAO circulating levels [65]. Consistent with this, a recent study in healthy humans consuming four eggs daily showed no significant increase in TMAO levels whereas consumption of comparable levels of choline provided as a choline bitartrate supplement, which is readily available to bacteria, raised TMAO levels significantly and augmented platelet reactivity [66].

## 6 Metabolites derived from amino acids

Proteolytic digestion and amino acid absorption are relatively efficient processes in the small intestine, but approximately 3–12 g of undigested protein is available for colonic bacteria [67]. Protein fermentation in the gut has the potential to be detrimental for the host as it generates multiple potentially toxic metabolites, such as phenolic compounds [68,69]. For instance, bacterial production of phenyl derivates by gut bacteria is associated with atherosclerosis and chronic kidney disease (CKD) [70,71].

### 6.1 Tryptophan

Tryptophan is an essential amino acid that cannot be produced by the human body and must be obtained through dietary proteins [72]. A small portion (about 1%) of ingested tryptophan is used for protein synthesis [73]. The majority of tryptophan is metabolized to a variety of biologically active compounds in different tissues, including serotonin, kynurenines, NAD+, tryptamine, and indoles. After protein digestion, a small fraction of tryptophan is used in the gut, whereas the majority of tryptophan is metabolized in the liver. Tryptophan and its liver products are then distributed to peripheral tissues, including the heart, muscle and brain, and exhibit numerous physiological functions.

**Figure 3:**
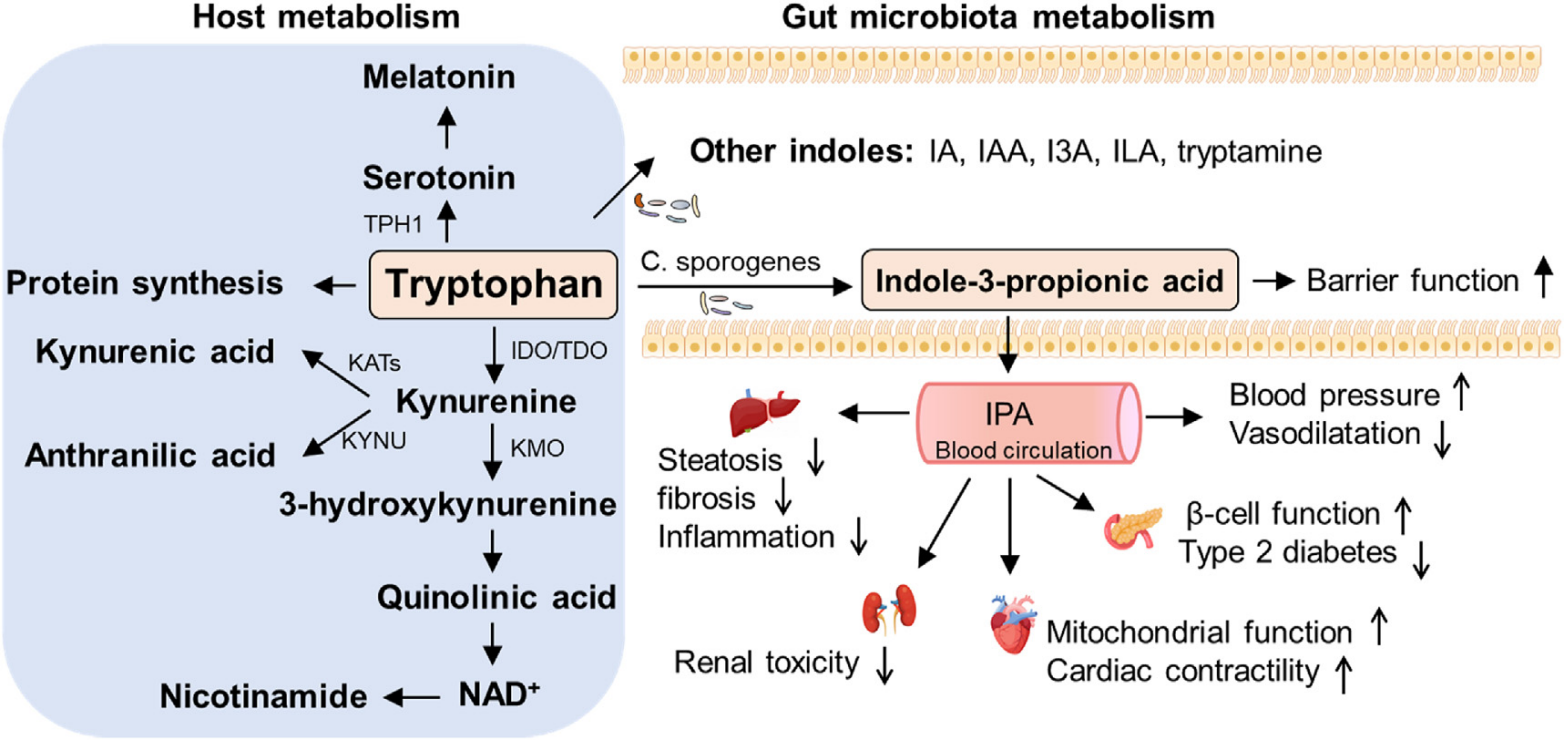
Tryptophan metabolites. Shown are metabolic pathways for tryptophan and its products mediated by host enzymes (left) or bacterial enzymes (right). IPA, in particular, has been studied for effects on intestinal traits and various cardiometabolic outcomes (see text).

Tryptophan metabolism is substantially affected by the gut microbiome (Figure 3). Gut bacteria metabolize tryptophan and thus regulate its availability to the host (Table 1). Selected gut bacteria metabolize tryptophan into indole and its derivatives, such as indoleacrylic acid (IA), indole-3-acetic acid (IAA), indole-3-aldehyde (I3A), indole-3-lactic acid (ILA), and indole-3-propionic acid (IPA) [74,75]. IAA is formed from indole-acetamide by bacteria that express indoleacetaldehyde dehydrogenase, including *Clostridium*, *Bifidobacterium*, and *Bacteroides* [76]. Tryptophan can be converted to I3A [77] and ILA by *Lactobacillus spp.* and *Bifidobacterium spp*. [78], respectively. When *Clostridium sporogenes* is present, tryptophan can be converted to IPA [75]. In addition to the small intestine, a certain amount of tryptophan can reach the large intestine, where it can be decarboxylated into tryptamine by commensal bacteria expressing tryptophan decarboxylases (TrpDs) [79].

In the gut, IPA binds to pregnane X receptors (PXRs) and maintains mucosal homeostasis and barrier function [80]. IPA also increases the expression of tight junction proteins including claudin-1, occludin, and ZO-1 [81,82]. Furthermore, IPA strengthens the mucus barrier by increasing mucins (MUC2 and MUC4) and goblet cell secretion products (TFF3 and RELMβ) and reduces the expression of LPS-induced inflammatory factors [82]. In addition, IPA modulates gut microbiota composition, inhibits endotoxin production and improves intestinal barrier in the gut, which is associated with protection against high-fat diet induced non-alcoholic steatohepatitis (NASH) [81].

IPA has also been shown to have direct protective effects systemically (Table 1). For example, IPA has been found to modulate mitochondrial function and improve the cardiac contractile function of isolated mouse hearts [83]. IPA increases blood pressure and cardiac contractility in normotensive rats, and increases the metabolic activity of cultured cardiomyocytes [84]. These observations are consistent with studies showing that IPA, in conjunction with the pregnane X receptor (PXR), reduces endothelium-dependent vasodilatation through suppression of eNOS-generated NO production [85]. In metabolic disorders, elevated plasma IPA concentrations were correlated with a lower risk of T2D [86,87], and reduced proportions of IPA producing bacteria were found in patients with T2D. While the mechanistic details for these associations have yet to be determined, clinical studies have shown that the protective effect of IPA could be related to insulin sensitivity and pancreatic beta-cell function [88].

### 6.2 Phenylalanine

Like tryptophan, phenylalanine is another essential amino acid obtained solely through dietary sources [89]. The association of phenylalanine with human disease is well-documented through the discovery of phenylkenoturia (PKU) nearly 100 years ago due to deficiency of phenylalanine hydroxylase [90]. The resulting elevation of circulating phenylalanine levels leads to neurotoxicity and intellectual disability, although it can generally be prevented by restricting protein intake from an early age. The majority of phenylalanine is absorbed in the small intestine whereas the small fraction that reaches the large intestine can be metabolized by gut bacteria to phenylpyruvic acid. Interestingly, phenylpyruvic acid was the compound first identified as being present in high concentrations in the urine of PKU patients [91] since it can also be generated from excess phenylalanine in the liver by endogenous l-amino acid oxidases.

Nemet and colleagues [92] recently compared the plasma metabolomics profiles of ∼1200 subjects from the GeneBank study with and without T2D to identify an unknown analyte that was associated with major adverse cardiac events (MACE = myocardial infarction [MI], stroke, or death). Targeted and quantitative MS methods unambiguously identified the metabolite as phenylacetylglutamine (PAG) and its adverse association with MACE was further validated in an independent, nonoverlapping set of ∼4000 GeneBank subjects. For example, both diabetics and nondiabetics in the highest quartile for PAG levels had ∼2-fold higher risk of incident MACE than subjects in the first quartile [92]. Circulating PAG levels (or phenylacetylglycine in mice) were markedly suppressed after administration of poorly absorbed oral antibiotics, demonstrating the obligate contribution of gut bacteria to production of PAG via the intermediate metabolite phenylacetic acid (PAA). Thus, these observations revealed a metaorganismal pathway where dietary phenylalanine is metabolized in the gut to an intermediate common to both mice and humans (PAA) that is absorbed and subsequently conjugated preferentially with either glutamine (humans) or glycine (mice) in the liver (Table 1).

Follow-up functional studies demonstrated PAG enhanced platelet responsiveness *in vitro* and promoted thrombus formation in a mouse model of carotid artery injury [92] (Table 1). Notably, colonization of gnotobiotic mice with a genetically engineered strain of *C. sporogenes* lacking one of the enzymes (*fldH*) that would shunt phenylalanine metabolism towards PAA increased thrombotic potential and decreased the time to cessation of blood flow in the carotid artery injury model. Further mechanistic studies demonstrated that the platelet activating properties of PAG are mediated through G-protein coupled receptors. Based on the structural similarity of PAG to catecholamines, such as isoproterenol and epinephrine, the authors hypothesized that PAG exerts its biological effects through adrenergic receptors (ADRs). Consistent with this notion, genetic and pharmacological perturbation experiments provided evidence that PAG promotes platelet aggregation *in vitro* and thrombus formation in mice via activation of ADR isoforms present on platelets (α2A, α2B, and β2). Interestingly, PAG levels were higher in urine samples from centenarians relative to those from non-centenarians, suggesting that increased excretion of PAG was associated with longevity. This may imply that PAG has pleiotropic effects that differentially modulate multiple metabolic processes and pathophysiologies [93]. Taken together, these studies provided another elegant demonstration of the power of untargeted metabolomics combined with common natural variation to identify novel pathways associated with cardiometabolic risk through interactions between dietary phenylalanine, gut bacteria, and host metabolism.

### 6.3 Histidine

As one of the least abundant amino acids [94], histidine is a nutritionally essential amino acid with unique chemical and metabolic properties such as containing a imidazole ring that functions in pH buffering, metal ion chelation, and antioxidation [[95], [96], [97], [98]]. There are common and unique pathways of histidine metabolism. Histidine can be converted to glutamate, and then oxidized to a-ketoglutarate by glutamate dehydrogenase [99]. In addition to protein turnover and glutamate generation, some unique metabolic pathways, including carnosine and histamine synthesis, make histidine an essential nutrient and beneficial for a number of disorders.

Histidine can be microbially converted to imidazole propionate (Table 1), a metabolite that is present at higher concentrations in patients with T2D and impairs glucose tolerance and insulin signaling at the level of insulin receptor substrate [100,101]. Mechanistically, imidazole propionate activates p38γ MAPK, which promotes p62 phosphorylation and subsequently activates mTORC1 [100]. In the heart, imidazole propionate protects against radiation-induced lung and heart toxicity, thus attenuating systolic dysfunction [102].

Human *Lactobacillus reuteri* strains contain the *hdc* gene cluster, including the histidine decarboxylase (HDC) and histidine-histamine antiporter genes, and convert histidine to histamine in the gut. Histamine is an important regulator of gastrointestinal functions, such as immune responses, gastric acid production, and mucosal ion secretion. Histamine activates histamine H2 receptor and suppresses intestinal inflammation in a mouse colitis model [103]. There are four G protein-coupled histamine receptors in humans and the predominant receptors in the intestinal epithelium are H1R and H2R [104,105].

### 6.4 Tyrosine

Tyrosine is another non-essential amino acid that serves as the precursor to various products, including neurotransmitters, hormones, alkaloids, phenols, and pigments. Phenols, such as p-cresol, are microbial metabolites of tyrosine [106,107] and have reported to increases paracellular permeability and reduces epithelial barrier function in a concentration-dependent manner [108,109]. Similarly, p-cresol exhibits cytotoxicity and reduces endothelial barrier function [109,110]. P-cresol sulfate, a sulfate-conjugate of p-cresol, has also been found to suppress contact hypersensitivity response (Th1-type cellular immune responses) and protect against allergic airway inflammation in mice [111,112]. In addition, increased p-cresol sulfate levels have been associated with CKD [113,114] (Table 1).

### 6.5 Branched-chain amino acids (BCAAs)

The BCAAs (leucine, valine and isoleucine) are also essential amino acids abundant in the diet. Among other emerging microbiota-derived compounds, BCAAs serve as important nutrients in bacterial physiology and have multiple functions. Elevated circulating BCAA levels have been found to be associated with obesity, insulin resistance, T2D [115,116] and risk of certain cancer [117,118]. Similarly, Ottosson et al. found that BCAAs were positively associated with BMI (body mass index), HOMA-IR (homeostatic model assessment for insulin resistance), and the abundance of *Blautia* [119]. The depletion of species belonging to the *Bacteroides* genus in obese individuals has been associated with higher levels of circulating BCAAs [120]. Additionally, cecal BCAA levels were higher in mice transplanted with gut bacteria from an obese human, suggesting that microbiota of obese individuals may have a higher capacity for production of BCAAs [121]. Furthermore, obese mice supplemented with *Bacteroides dorei* and *Bacteroides vulgatus* exhibited improved BCAA catabolism in brown adipose tissue and attenuated body weight gain [122]. Together, these studies suggest that variation in intestinal microbiota may represent a potential mechanistic link between BCAA metabolism, and metabolic disease (Table 1).

## 7 Metabolites derived from sterols

**7.1 Primary and Secondary Bile Acids**

Dietary lipids represent another class of nutrients that can affect systemic host health, gut microbial composition, and derived circulating metabolites [31,123]. For example, germ-free mice exhibit altered cholesterol metabolism, and studies have shown that gut microbiota influences cholesterol metabolism [6], in part through regulating cholesterol biosynthesis in the liver [124]. Human and animal studies also indicate that probiotics can decrease systemic blood lipids by redistributing cholesterol from plasma to the liver and/or by affecting gut cholesterol absorption [125], although how probiotics decrease cholesterol levels remains poorly understood. Recently, Kenny et al. reported that coprostanol-forming bacteria can potentially decrease cholesterol absorption in the intestine resulting in lower serum levels [126].

Another aspect of the relationship between the microbiome and sterols are bile acids (BAs) (Table 1). For example, primary BAs in the form of cholic acid, chenodeoxycholic acid, and, to a lesser extent, hyocholic acid are synthesized in the liver from cholesterol and conjugated to glycine or taurine [127]. Conjugated BAs are stored in the gallbladder and secreted into the small intestine upon ingestion of a meal, where they help emulsify and solubilize fats and facilitate the transport of nutrients. While most are reabsorbed in the distal small intestine and returned to the gallbladder, a small amount (∼5%) of BAs enters the colon where they are subjected to structural modifications by gut microbes through deconjugation-, dehydrogenation, and dihydroxylation to generate deoxycholic acid, lithocholic acid, hyodeoxycholic acid, and many other so-called secondary BAs [127]. Deconjugation of host-derived primary BAs occurs via bile salt hydrolases (BSH), which are widespread in bacteria. Bile salts may provide nutrition for bacteria by liberating amino acids and could aid in the incorporation of cholesterol and bile components in the bacterial membranes. The hydroxygenation reactions are carried out by three distinct hydroxy steroid hydroxygenases, 3α-, 7α-, and 12α-, which result in the oxidization of specific hydroxyl groups on BAs. There has been speculation that the diverse metabolites resulting from these reactions may act as signaling molecules for microbes to communicate with each other or alter host physiology. 7α-dehydroxylation, is only known to be performed by a small number of anaerobic species. Since BAs can be toxic to bacteria, not only can gut microbes influence BA pools, but BA pools can conversely affect the structure of gut microbial communities.

Other potential mechanisms through which BAs are thought to influence cardiometabolic pathways may be related to their direct activation of cellular receptors, such as the nuclear receptor farnesoid X receptor (FXR) and the membrane Takeda G-protein coupled receptor 5 (TGR5) [128]. Most of our understanding on how BAs influence cardiometabolic traits comes from *in vitro* studies, the use of genetically modified animal models, or indirectly through the use of FXR and TGR5 agonists/antagonists. For example, FXR and TGR5 have been shown to regulate glucose, lipid, and energy metabolism [[129], [130], [131], [132], [133]], vascular wall inflammation [[134], [135], [136]], atherosclerosis [137,138], and cardiac function [139,140].

Although technical difficulties and biological differences between mice and humans have hampered progress in this area to some extent, limited clinical associations between circulating BAs and cardiometabolic outcomes in human cohorts have been reported. For example, a small clinical trial with the secondary BA, ursodeoxycholic acid (UDCA), demonstrated improvements in liver function scores [141]. In a more recent study, levels of glycoursodeoxycholic (GUDCA, the glycine-conjugated form of UDCA) were lower in serum and stool of hyperglycemic subjects, and ameliorated diet-induced insulin resistance and hepatic steatosis when fed to in mice [142]. However, BA composition differs significantly between humans and mice, including their conjugation to glycine (humans) or taurine (mice). These observations suggest that mouse studies investigating the interactions between gut microbiota, host BA metabolism, and cardiometabolic traits may need to be interpreted with caution if being used to gain insight into human diseases [143]. The varying affinities that different BAs have towards FXR and TGR5 further complicate efforts to a better understanding of the role of these metabolites in cardiometabolic disease [[144], [145], [146], [147]]. Lastly, studies evaluating total BA levels by enzyme immunoassays for association with CAD have been inconsistent [148,149]. Some of these challenges may be overcome with technological advances, such as the recent development of quantitative MS assays to simultaneously measure a large panel of primary and secondary BAs in human plasma [150].

## 8 Microbiome-accessible carbohydrates (MACs)

### 8.1 Short-chain Fatty Acids (SCFAs)

SCFAs are among the most abundant and most highly studied gut microbial metabolites. They are produced by anaerobic fermentation of carbohydrates reaching the distal gut, including dietary fiber and prebiotics [12,151]. The main SCFAs in the gut are acetate, propionate, and butyrate (Table 1), which are produced at a ratio of ∼60:20:20, respectively, and a combined luminal concentration of ∼100 mM [12,152]. However, establishing accurate concentrations of SCFAs in the gut or feces is difficult considering the dynamic nature between substrate availability, bacterial production, and colonic absorption. Nonetheless, some of the beneficial properties of dietary fiber can be attributed to its modulation of the gut microbiome and production of these beneficial SCFAs. For example, studies have shown that SCFAs can modulate epithelial turnover and strengthen tight junctions [153]. In particular, butyrate influences epithelial oxygen consumption and stabilizes hypoxia-inducible factor, which is critical for maintenance of epithelial barrier function [154].

Once produced in the intestinal lumen, SCFAs are transported into colonocytes, where they serve as substrates for energy and metabolism. Butyrate induces intestinal glucose production by regulating gluconeogenic gene expression whereas propionate serves as a substrate for intestinal gluconeogenesis [155]. The remaining SCFAs are shuttled to the liver via the portal vein where acetate, for example, is integrated into the circulating acetate pool, which consists of both bacterially-derived and host-produced acetate [156]. SCFAs are mainly excreted through feces, lungs and, to a less extent, via urine [151,157,158]. Although SCFAs concentrations are present in peripheral blood at significantly lower levels compared to the gut and portal circulation, circulating butyrate levels are inversely related to endotoxemia and systemic inflammation [159].

SCFAs have been shown to modulate several cardiometabolic health traits including lipid metabolism, glucose homeostasis, inflammation, and atherosclerosis. In diabetic rats, butyrate supplementation led to a reduction in serum cholesterol and triglycerides and improved glucose tolerance [160]. A clinical trial with T2D patients demonstrated that dietary supplementation of butyrate and inulin, a type of dietary fiber, resulted in improved glycemic homeostasis [161]. Furthermore, bidirectional Mendelian randomization (MR) analysis revealed that host genetic-driven increase in gut production of butyrate was associated with improved insulin responses in a large human cohort of normo-glycemic individuals [162]. These observations are consistent with the lower levels of butyrate-producing bacteria displayed by cardiometabolic disease patients compared to healthy individuals [163]. Several studies have demonstrated that SCFAs can also have anti-inflammatory effects [164]. Indeed, immunomodulation is associated with many of the cardiometabolic benefits of SCFAs. For example, Aoki et al. demonstrated that bacterially-produced acetate ameliorated non-alcoholic fatty liver disease and suppressed hepatic inflammation in mice [165]. In addition, studies by Kasahara and colleagues [166] revealed that the prominent gut-associated butyrate producing bacterial genus *Roseburia* was negatively correlated with atherosclerosis. Gnotobiotic studies subsequently implicated butyrate in protection against aortic lesion development [166]. More recently, an atheroprotective effect of propionate has also been reported [167]. For example, in mice, propionate was shown to attenuate aortic lesion formation and lower plasma total cholesterol and LDL cholesterol levels, which could be attributed, in part, to decreased expression of intestinal cholesterol absorption genes [167]. Notably, a small 8-week clinical intervention with propionate in healthy volunteers also led to a more favorable lipid profile.

Although circulating concentrations of SCFAs, especially propionate and butyrate, are significantly lower relative to their intestinal levels, SCFAs demonstrate systemic effects on host metabolism [164]. There are four known G protein-coupled receptors that sense SCFAs (GPR41, GPR43, GPR109a, Olf78), each of which exhibit differing affinities for each SCFA and present unique tissue expression patterns [168]. Although they are all expressed in the intestine, these receptors are also expressed in peripheral tissues, including the pancreas, adipose, spleen, and various immune cells [168] Circulating concentrations of SCFAs tend to be lower than the reported half-maximal effective concentrations of these receptors suggesting that low-level activation may be sufficient to trigger physiological effects. SCFAs are also known to reduce systemic inflammation by promoting mucosal health and improve gut barrier function [164], thus limiting translocation of proinflammatory material such as lipopolysaccharide (LPS) from the intestine into systemic circulation.

## 9 Summary and future prospects

Population-based integrative approaches have identified a large number of microbe-derived or microbe-modified metabolites that are associated with cardio-metabolic traits, and many of these have been verified by experimental approaches such as the use of germ-free mice. It is important to note that, because population-based genetics approaches only generate hypotheses, direct experiments are essential to validate the causal relationships. The metabolites in some cases act directly on intestinal cells but, in most cases, enter the host circulation and interact with specific target cells and tissues. In many cases, the mechanisms by which the metabolites exert their effects are still unclear. An important problem in human studies is the inability to obtain tissue samples other than blood. Studies in mouse populations offer the advantage that omics data can be collected from relevant tissues and that the environments can be controlled [35]. Two important additional applications of such integrative approaches are to determine how the gut microbiome composition is influenced by host genetic variation and by diet/lifestyle. However, the mapping power of 100 inbred strains in the HMDP is limited as compared with human GWASs with thousands of samples. Meta-analysis incorporating data from traditional crosses or more inbred and recombinant inbred strains can be used to improve power [[169], [170], [171]]. Of note, it is essential to correct population mapping to avoid false positive associations. This can be accomplished by mixed model algorithms including EMMA and FaST-LMM [172,173]. Simulation, a Bonferroni correction, or a false discovery rate can be used to determine genome-wide significance [174,175].

An important aspect of systems-based approaches is the ability to integrate multiple levels of data. Studies with mouse populations provide an advantage since omics data can be collected directly from tissues relevant to cardiometabolic outcomes, such as the vasculature, liver, adipose, and intestines. Such data combined with genetic variation, gut bacterial composition, and clinical phenotypes allows correlation, co-mapping, or various modeling analyses to be carried out, which can generate more directed hypotheses regarding the relationships between the various levels of data. This approach was illustrated by Org and colleagues in an HMDP study where coincident genome-wide significant association of *Roseburia* spp. abundance and subcutaneous fat mass were observed with a locus on chromosome 15. Because of the availability of transcriptomics data in adipose and liver tissues, the authors were further able to show that the same locus yielded expression quantitative trait loci (eQTL) for several positional candidate genes. Furthermore, of the positional candidates at this locus, mRNA levels of *Irak4*, which is involved in the innate immune response, were correlated with both the abundance of *Roseburia* spp. and insulin resistance, suggesting a causal relationship with this gene [34]. Similar approaches have also been applied to other mouse populations, including advanced intercross lines, the collaborative cross, and diversity outbred mice [35]. By comparison, the gold standard for establishing causality in humans is the randomized clinical trial, which can be challenging and expensive to carry out. More recently,

Mendelian randomization (MR) with genetic data is another strategy that has proven useful for establishing causal relationships. For example, several MR studies have suggested that various bacterial taxa or gut microbiome-derived metabolites are causally associated with cardiometabolic traits [162,176]. However, validation in additional studies with larger sample sizes will be needed before such causal relationships are firmly established. Some cohorts, such as the Metabolic Syndrome in Men (METSIM) study, a population-based study of ∼10,000 Finnish men, are promising in this regard since multi-omic data are available [177]. Despite its strengths, systems studies with omics data are still comprehensive screens for associations between various levels of data (i.e., gut bacteria, metabolites, clinical traits) that are not necessarily quantitative. Thus, it is critical to verify any identified associations with independent methods and validation cohorts to avoid potential false positive signals. In this regard, using an appropriate statistical threshold that considers the large number of comparisons made in systems studies is one important component to using rigorous experimental designs.

It is important to note that in addition to the vast metabolic diversity observed in the gut microbiome, it is also becoming evident that there is a high degree of functional redundancy [178]. For example, it is uncommon that a single bacterial strain or even a species to be responsible for production of a metabolite [56,63]. Thus, microbiome analyses should consider candidate microbial pathways, ideally expression levels (mRNA or protein) of the genes contributing to production of bioactive metabolites, rather than just quantitation of the abundance of taxa previously described to have such capabilities. Furthermore, functional predictions based on sequence homology should be considered as hypotheses and require direct experimental confirmation, as it is becoming clear that the presence of a gene encoding a specific enzyme does not necessarily result in such enzymatic activity being expressed [63].

Given the associations between gut microbiota and cardiometabolic outcomes, developing new therapeutic strategies targeting the gut microbial communities is a promising approach to prevent or treat these diseases. Considerable effort into developing strategies has been used to achieve this goal [179], including prebiotics [180,181], probiotics [[182], [183], [184]], fecal microbiota transplantation [185,186], metabolites [[187], [188], [189], [190], [191]], phages [192,193], miRNAs [194], and hyaluronan [195,196]. In particular, studies in mice have demonstrated that transplantation of cecal microbes from an atherosclerosis-prone strain with high TMA/TMAO levels to a low TMAO-producing and atherosclerosis-resistant strain can enhance production of these pro-atherogenic metabolites with a corresponding increase in aortic lesion formation [197]. These observations raise the intriguing question of whether using the converse strategy with bacterial transplantation from individuals with low TMA/TMAO-producing capacity, such as vegans [53], can decrease risk of CAD. Although such approaches have yet to be implemented in humans with respect to CAD, fecal transplantation studies have shown that transfer of gut microbes from lean donors through a duodenal infusion into patients with metabolic syndrome can improve insulin sensitivity [198]. In another strategy, a small molecule choline analog, 3, 3-dimethyl-1-butanol (DMB), was designed to competitively inhibit diverse and phylogenetically distant classes of microbial TMA lyases, which were previously identified as enzymes that catalyze the conversion of choline to TMA [51,199]. Chronic feeding of DMB to mice in the context of a high-choline diet led to shifts in the proportions of some bacterial taxa and substantial reductions in plasma TMAO levels, macrophage cholesterol accumulation, foam cell formation, and atherosclerotic lesions, without any evidence of toxicity or adverse cardiometabolic effects in the animals [200]. Lastly, another promising approach may be to remodel gut bacterial composition through the use of cyclic D, l-α-peptides. For example, chronic oral administration of several different cyclic peptides to hyperlipidemic atherosclerosis-prone mice shifted the gut microbiome from a high-fat state to one resembling a chow diet and reduced cholesterol levels and aortic lesion formation [201]. Taken together, these results suggest that targeting gut microbial composition or production of bacterial-derived metabolites through transplantation or specific pharmacological manipulation may serve as potentially novel therapeutic approaches for treating cardiometabolic disease.
